# A Preliminary Analysis of a Modified Anterior Approach to Hip Pericapsular Neurolysis for Inoperable Hip Fracture Using the IDEAL Framework

**DOI:** 10.3390/healthcare10061002

**Published:** 2022-05-28

**Authors:** Tony Kwun-Tung Ng, Jui-An Lin, Sumire Sasaki

**Affiliations:** 1 Pain Management Unit, Department of Anaesthesia and Operating Theatre Services, Tuen Mun Hospital, 23 Tsing Chung Koon Road, Tuen Mun, Hong Kong, China; tonyktng@gmail.com; 2 Department of Anaesthesiology, LKS Faculty of Medicine, The University of Hong Kong, Pokfulam, Hong Kong, China; 3 Department of Anaesthesia and Intensive Care, Faculty of Medicine, The Chinese University of Hong Kong, Shatin, Hong Kong, China; 4 Center for Regional Anesthesia and Pain Medicine, Wan Fang Hospital, Taipei Medical University, Taipei 116, Taiwan; 5 Department of Anesthesiology, Wan Fang Hospital, Taipei Medical University, Taipei 116, Taiwan; 6 Department of Anesthesiology, School of Medicine, College of Medicine, Taipei Medical University, Taipei 110, Taiwan; 7 Pain Research Center, Wan Fang Hospital, Taipei Medical University, Taipei 116, Taiwan; 8 Department of Anesthesiology, School of Medicine, National Defense Medical Center, Taipei 11490, Taiwan; 9 Department of Anaesthesia and Operating Theatre Services, Kwong Wah Hospital, 25 Waterloo Road, Yau Ma Tei, Hong Kong, China; sumire.sasaki@gmail.com

**Keywords:** hip fractures, hip joint, neurolysis, pain management, IDEAL classification

## Abstract

**Introduction:** With an increasingly ageing population, there is a growing impact of fragility hip fracture on the healthcare system and on society as a whole. Oral and injectable analgesics are often insufficient whilst traction and regional blocks do not allow patients to be discharged easily. While the conventional approach of ultrasound-guided anterior hip pericapsular neurolysis can help a lot of inoperable hip fracture patients to relieve their fracture pain and facilitate subsequent nursing care, enormous technical challenges are encountered in some cases. In this retrospective case study, we evaluated the overall pain and functional outcomes of our modified approach of anterior hip pericapsular neurolysis for inoperable hip fractures using the IDEAL framework. **Method**: This retrospective case series studied patients with acute inoperable hip fracture who received the modified approach of anterior hip pericapsular neurolysis from January 2018 to June 2019 according to the IDEAL recommendations. The modified approach consisted of pericapsular nerve group (PENG) injection, iliopsoas plane infiltration, and the sagittal approach of obturator nerve articular branches (ONAB) injection. Subsequent alcohol neurolysis would be performed in the same setting if there were positive diagnostic blocks. Assessments were carried out on post-intervention day 5. The primary outcome was pain intensity during hip flexion at 80 degrees in the recumbent position and during gentle hip internal and external rotation using an appropriate pain scoring tool. The secondary outcomes were the range of tolerable hip flexion and occurrence of any lower limb neurological deficit because of the procedure. Interim outcomes were also briefly evaluated. **Results**: Among the 74 patients who were reviewed in the study period, the median dynamic pain at hip flexion 80° (*p* < 0.001) and on gentle hip external and internal rotation (*p* < 0.001) was significantly reduced from a composite score of 3 (severe pain) to 1 (mild pain) on post-intervention day 5 after the modified approach of hip neurolysis. This translated to 72% of patients achieving satisfactory pain control, which was defined as a composite pain score of ≤1 on hip flexion at 80°. Functionally, the mean range of tolerable hip flexion significantly improved from 39.7° at baseline to 74° on post-intervention day 5 (*p* < 0.001). Transient and reversible hypotension was seen in about 10% of the patients. No other major procedural adverse event was noted. Interim follow-up at 4–6 months post-intervention revealed that more than 95% of patients continued to have satisfactory dynamic pain control (i.e., composite pain score ≤ 1). According to the IDEAL classification, this study could be ranked as stage 2a (development). **Conclusions**: Our findings suggested that anterior hip pericapsular neurolysis using a modified approach could offer consistent and satisfactory analgesic and functional benefits to a majority of patients with inoperable hip fractures during the interim of the fracture healing process, and it was potentially safer than the conventional approach. This technique might have achieved its readiness to proceed to the next stage of research according to the IDEAL framework.

## 1. Introduction

**Figure 1:**
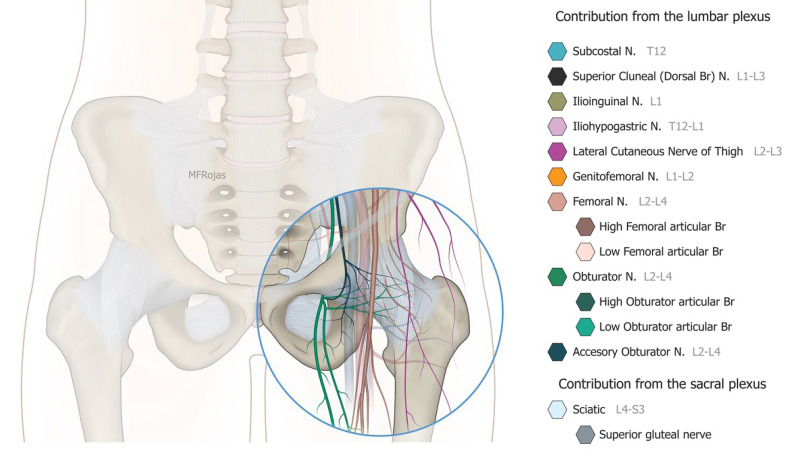
Nerves supplying the hip joint and their relationship to the femoral vessels. Reproduced with permission from Dr Maria Fernanda Rojas Gomez (Bucaramanga, Colombia).

The growing impact of fragility hip fracture on the healthcare system and on society has become a major concern worldwide. Despite the advances in perioperative medicine and anaesthetic care, a minority of frail patients are still too ill to be surgical candidates. Hip fracture analgesia generally includes non-pharmacological modalities, oral and injectable analgesics, traction and regional nerve block [1,2]. However, given the complexity of medical comorbidities in this patient population, pain management of hip fracture is not a one-size-fits-all recipe [1]. Acupuncture, relaxation therapy, and transcutaneous electrical nerve stimulation (TENS) may be associated with potentially clinically meaningful benefits, but no firm conclusion can be drawn from the current evidence [1]. Comorbidities may also prohibit the use of these therapies, for instance, pacemakers and anticoagulants. Simple non-opioid analgesics are often insufficient to treat dynamic pain adequately, whereas opioid analgesics can cause disturbing side effects in this patient population, such as dizziness, nausea and vomiting, sedation and even respiratory depression. Traction does not reduce the intensity of acute pain, and patients also remain bed-ridden during this treatment [2]. Regional nerve blocks, such as Pericapsular Nerve Group (PENG) block, fascia iliaca block and femoral nerve block, can effectively manage fracture pain, especially if a catheter is inserted [1,2,3]. Nevertheless, the benefit from a single shot regional nerve block can usually last for 1 to 3 days whilst there is an infective concern if a block catheter is placed for weeks. Community management of the catheter is also a practical concern in our locality. Partial hip denervation hence becomes a treatment option to provide long-lasting analgesia by a single intervention. With the sophistication of ultrasound technology coupled with a greater understanding of hip joint innervations in recent cadaveric studies (Figure 1), there has since been a venture to develop an ultrasound-guided approach to denervate the hip joint. The first such approach was described by Sasaki et al. in 2018 [4], where alcohol was infiltrated superficially over the ligaments encasing the anterior hip joint, namely the iliofemoral and pubofemoral ligaments. Subsequent to our case series demonstrating the efficacy of the conventional approach, which consisted of a PENG injection and an oblique approach to the obturator nerve articular branches (ONAB) [5], we noticed there were circumstances in which the conventional approach did not work well, and enormous technical difficulties were encountered in terms of needle trajectory.

We hence developed a novel modified approach to tackle these clinical challenges in late 2017. From January 2018 onwards, as a standard institutional practice, we fine-tuned our technique of anterior hip pericapsular neurolysis that was originally published in our first case series [5]. The modified approach highlighted two differences compared with our classical approach, which referred to an addition of pericapsular infiltration in the iliopsoas plane to cover the low articular branch of the femoral nerve and a sagittal approach to target the ONAB. The objectives of this study are to retrospectively evaluate the analgesic and functional outcomes of our novel modified approach of anterior hip pericapsular neurolysis in inoperable hip fracture patients from January 2018 to June 2019 according to the IDEAL (idea, development, exploration, assessment, long-term study) framework.

## 2. Materials and Methods

### 2.1. Study Design

This was a preliminary retrospective analysis of the outcomes of a modified approach of alcohol neurolysis of the femoral nerve articular branches (FNAB), accessory obturator nerve, and the obturator nerve articular branches of the hip joint in the format of case series. The study design followed the IDEAL framework.

### 2.2. Patient Population

Hip fracture patients were evaluated by an anaesthetist and an orthopaedic surgeon. In the case that the patients were considered as too risky for operative management, conservative management would be considered with referral to the pain management unit in our institution. From January 2018 to June 2019, all such patients presenting to the pain management unit were evaluated for hip neurolysis. This treatment option was based on our previous satisfactory experience [1,2]. To be eligible for neurolysis, the patients needed to fulfil the following criteria: (1) radiographic confirmation of acute traumatic hip fracture (neck of femur or trochanteric) within 4 weeks; (2) moderate to severe hip fracture pain (both rest and dynamic) despite maximized conservative treatments (tractions, oral, or injectable analgesics); (3) unacceptable anaesthetic risks; (4) relatively low ambulatory potential as determined by an orthopaedic surgeon; (5) positive diagnostic block (≥50% in pain score reduction on hip flexion at 45 degrees). The exclusion criteria were: (1) inability to lie in a recumbent or semi-recumbent position; (2) hemodynamic instability; (3) >50% O2 supplementation or assisted ventilation; (4) previous ipsilateral hip neurolysis; (5) significant coagulopathy (platelet count <50,000/mL or international normalized ratio >1.5).

### 2.3. Consent

For eligible patients, written informed consent was sought from the patients, their powers of attorney, or legal caregivers subject to the cognitive status of patients. The informed consent process consisted of the purpose of the intervention, a brief explanation of how we performed the intervention, the pros and cons of this intervention, and the potential risks which included but were not limited to the following: treatment failure, procedural discomfort, wound infection, wound hematoma, vascular injury, transient neuritis, systemic side effects of alcohol injection and allergy. As per our institutional practice, this intervention was arranged as an emergency procedure in the hospital emergency operation list and was performed in either the operation theatre or in the regional block corner in our operation suite during office hours. It was performed by a pain specialist or anaesthesia specialist who was under pain medicine training, with the support from one or two pain nurses.

Institutional approval of the study was sought from the New Territories West Cluster (NTWC) Research and Ethics Committee, Hospital Authority, Hong Kong (ref no.: NTWC/REC/21063).

### 2.4. Intervention

**Figure 2:**
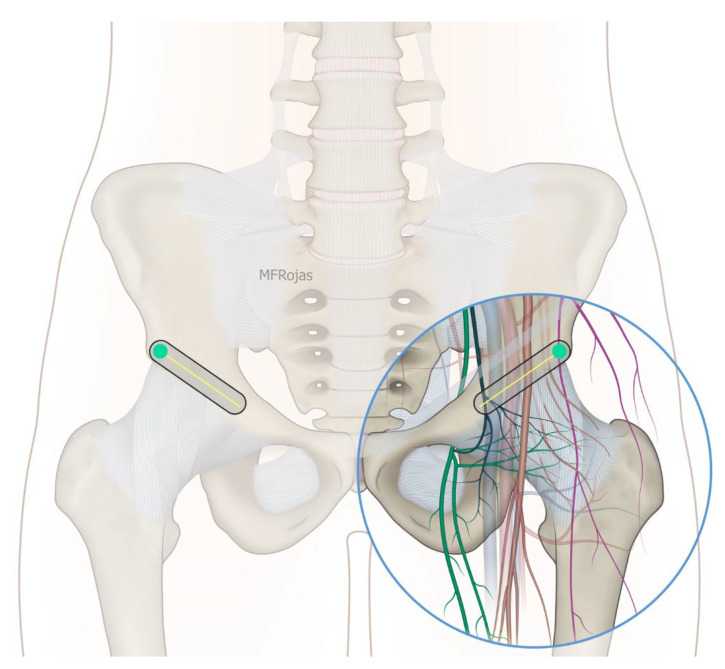
A schematic diagram to show how the linear probe is placed to align the AIIS and IPE. Reproduced with permission from Dr Maria Fernanda Rojas Gomez (Bucaramanga, Colombia). Green dot: transducer mark.

**Figure 3:**
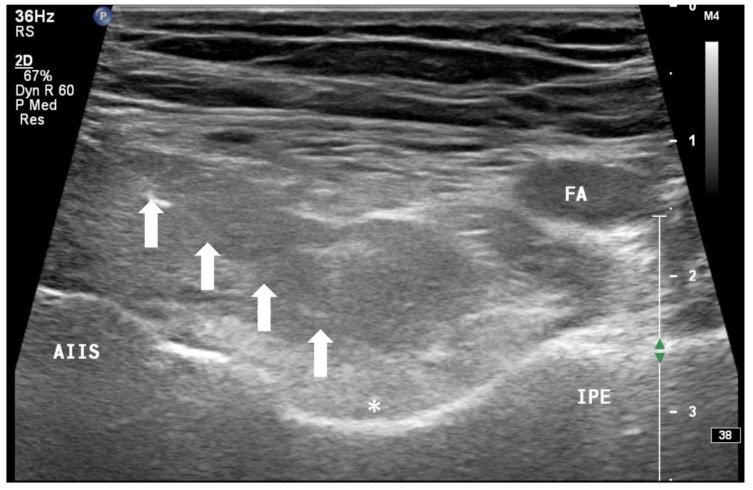
Ultrasound image showing injection of FNAB and AON by infiltration of the drug between the IPE and AIIS from lateral to medial (PENG approach). FA: femoral artery, AIIS: anterior inferior iliac spine, IPE: iliopectineal eminence, *: absolute alcohol spreading underneath iliopsoas muscle above the ASIS-IPE bone surface, white arrow: needle.

**Figure 4:**
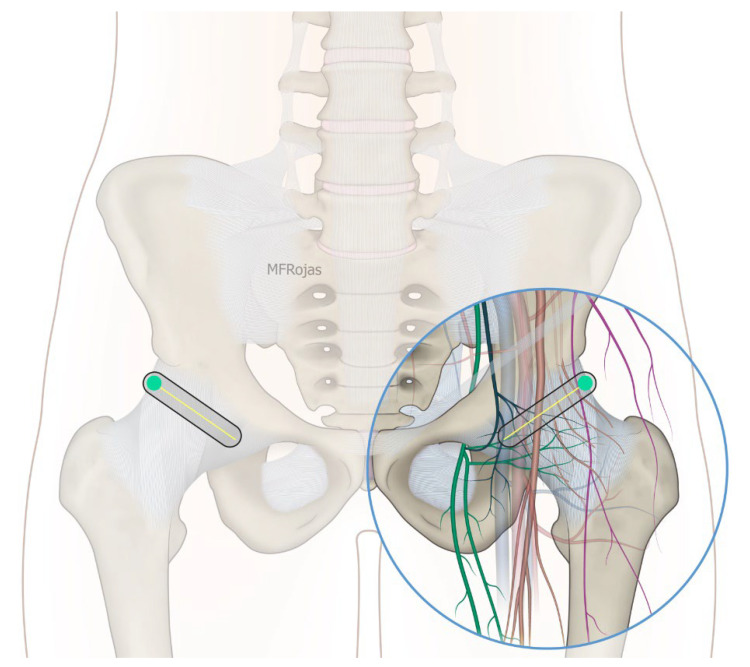
A schematic diagram to show that the probe slides caudally from the IPE-AIIS plane until the pubic bone drops out and the hypoechoic capsule is revealed. Reproduced with permission from Dr Maria Fernanda Rojas Gomez (Bucaramanga, CO). Green dot: transducer mark.

**Figure 5:**
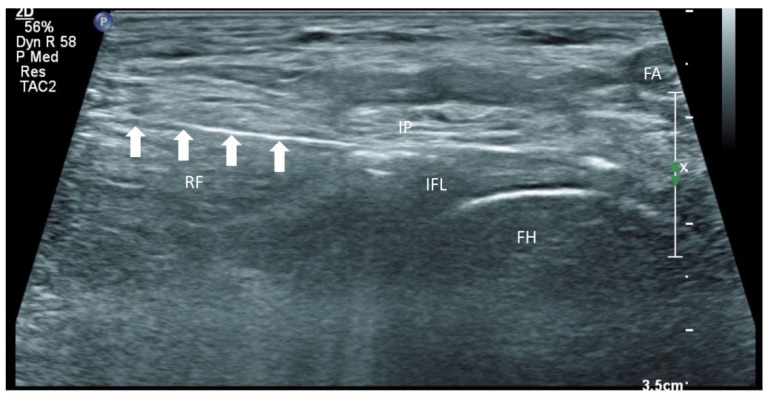
Ultrasound image showing the iliopsoas plane infiltration. FA: femoral artery, FH: femoral head, IP: iliopsoas, RF: rectus femoris, IFL: iliofemoral ligament; white arrow: needle.

**Figure 6:**
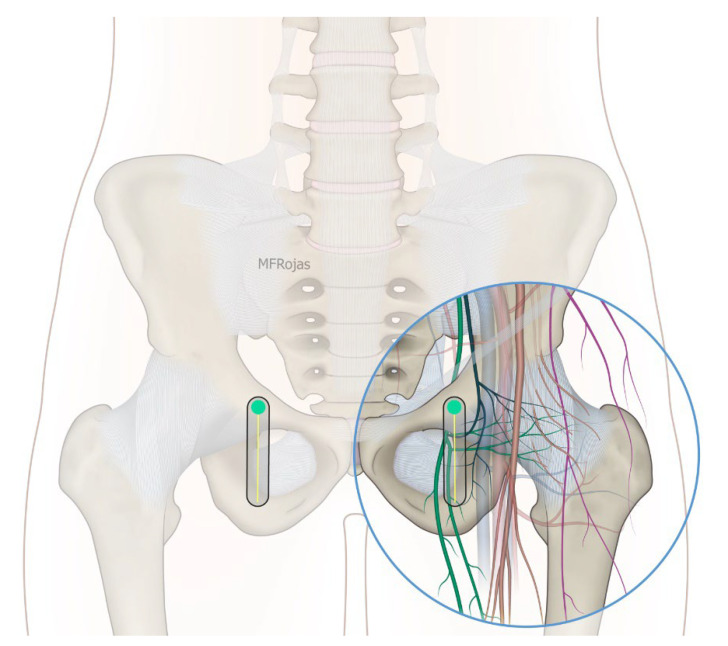
Schematic diagram showing the sagittal plane at the IMA with femoral vessels avoided. Reproduced with permission from Dr Maria Fernanda Rojas Gomez (Bucaramanga, CO). Green dot: transducer mark.

**Figure 7:**
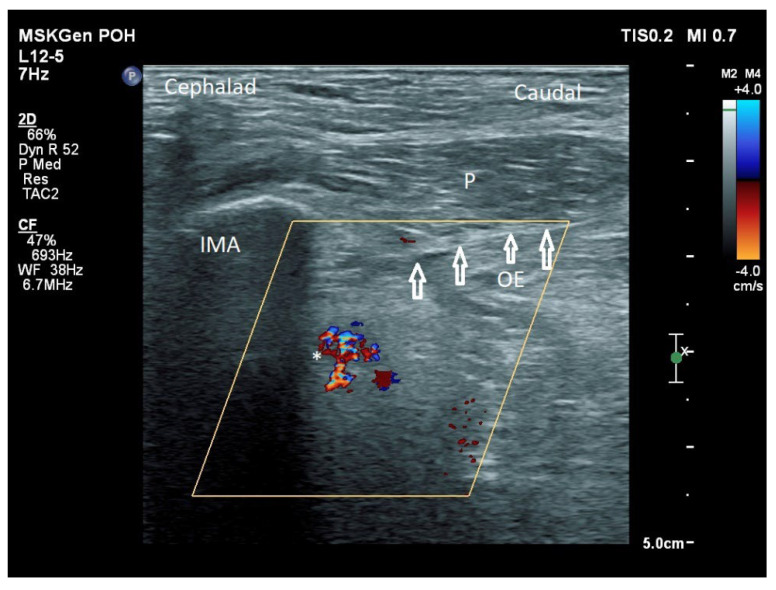
Approaching the ONAB in a sagittal plane from a caudal to cephalic direction. IMA: inferomedial acetabulum, P: pectineus, OE: obturator externus, *: acetabular and/or posterior branch of the obturator artery, white hollow arrow: needle.

In addition to the classical PENG injection, our modified approach highlighted the addition of pericapsular infiltration in the iliopsoas plane to cover the low articular branch of the femoral nerve and a sagittal approach to target the ONAB. The algorithm was still used to perform diagnostic blocks, followed by alcohol neurolysis 10 min later for those with a positive diagnostic block in the same setting under real-time ultrasound guidance (Philips Affiniti 70, 5–12 MHz linear probe or 1–5 Hz curved probe depending on the depth of the target). In the modified approach, the procedure consisted of 3 needle insertions with the use of 22-gauge 10 cm Quincke needles. The first needle for the high femoral nerve articular branches (high FNAB branches) and AON was inserted in the same way as the PENG approach between the anterior inferior iliac spine (AIIS) and the iliopubic eminence (IPE) deep to the psoas tendon (Figure 2 and Figure 3). A second needle for the low branches of FNAB was inserted in-plane from lateral to medial and advanced through the sartorius, rectus femoris and iliopsoas muscles. The final needle position was in the plane superficial to the iliofemoral ligament medial to the rectus femoris tendon over the femoral head (Figure 4 and Figure 5). The third injection for the obturator nerve articular branches was used to target the caudal aspect of the inferomedial acetabulum (IMA) by scanning in a sagittal plane with a caudal-to-cephalad needle trajectory. The precise localization of the caudal aspect of the IMA could be first approached via the ordinary way (an oblique scan) as described in our first case series [5]. The probe was slowly rotated obliquely until the acetabulum, femoral head, and neck were aligned in the same plane, with the superomedial acetabulum coming into view. The probe was then slid caudally with the end point being the presence of the acetabulum without the femoral head. This referred to the location of the inferomedial acetabulum. The probe was then adjusted until the IMA was in the middle of the image, followed by rotating the probe by approximately 60 degrees (Figure 6). In this scan, the pectineus, the caudal aspect of the IMA and, occasionally, the obturator externus were seen. The ONAB could be visualized in the wedge of the subpectineal space directly adjacent to the IMA. Before any needle insertion, the probe was tilted slightly medially towards the obturator foramen to look for the acetabular/posterior branch of the obturator vessels which are usually deeper than the ONAB. The needle was then inserted from caudal to cephalad in-plane until the needle tip reached the wedge space consisting of the ONAB (Figure 7). In case the wedge space could not be clearly visualized, the needle tip would then be advanced until touching the caudal bony surface of the IMA directly underneath the pectineus. Then, 0.5–1% ropivacaine (4, 3 and 2 mL, respectively) was injected into the above three landmarks. The needles were left in situ during the diagnostic test while their positions were reconfirmed by ultrasound before 100% alcohol was injected into the above targets with volumes in a 1:1 ratio between alcohol and local anaesthetics. A decrease of ≥50% in composite pain score on hip flexion at 45 degrees 10 min after the blocks signified a positive diagnostic block. As a post-neurolysis routine manner, stylets were reinserted before needle withdrawal.

### 2.5. Monitoring

Standard monitoring with electrocardiogram were routinely applied to all patients, and a functioning intravenous access was ensured before the intervention commenced. A pain nurse was responsible for monitoring the clinical status of patients and their vital signs. Resuscitation drugs (ephedrine, atropine and phenylephrine) and sedative drugs (midazolam) were on standby for every patient.

### 2.6. Follow-Up and Assessments

Following the procedure, the follow-up assessments were performed by pain nurses 20 min after the neurolytic procedure and on post-intervention day 5 at the hospital. We measured tolerable passive hip flexion with a goniometer, as well as pain intensity during hip flexion at 80 degrees in the recumbent or semi-recumbent position and upon gently rotating the hip internally and externally. We also documented any lower limb neurological deficit along the femoral nerve and obturator nerve distribution by checking the cold sensation to ice.

In this patient population, it is not uncommon to have cognitive impairment, leading to difficulty in applying a single pain assessment tool towards them. As such, the Numeric Rating Scale (NRS), Verbal Descriptive Scale (VDS), or Pain Assessment in Advanced Dementia Scale (PAINAD) was used when appropriate. While NRS and VDS are popular pain assessment tools, older adults preferred the VDS when given a choice [6,7,8,9,10,11]. The PAINAD was found to be an accurate and reliable assessment tool in the adult patients who were unable to report pain reliably and accurately because of their impaired cognitive functions [10,11]. This tool consisted of 5 items with numerical equivalents for each of the 5 behavioural items (i.e., breathing, negative vocalizations, facial expression, body language, and consolability), with the total score ranging from 0 to 10. Each of the 5 items contained a range from 0 to 2, and the summation of each of the 5 items constituted the total score. Since there was evidence to support positive correlation between the PAINAD and NRS, and the equivalency between the NRS and VDS [9,10,11], a composite pain score scale was developed from the above measurement tools to facilitate statistical analyses as what we performed in our previous publication [12]. As shown in Table 1, a composite pain score pf 0 was equal to no pain; composite pain score 1 (mild pain) meant NRS 1–3/10, PAINAD 1–3/10, and VDS slight and mild pain; composite pain score 2 (moderate pain) meant NRS 4–6/10, PAINAD 4–6/10, and VDS moderate pain; and finally, NRS 7–10/10, PAINAD 7–10/10, and VDS severe, very severe, and most intense pain possible were categorized as composite pain score 3 (severe pain).

The primary outcome measure was the dynamic pain score on hip flexion to 80 degrees on post-intervention day 5 compared with that before pain intervention. Subject to their cognitive status, one of the above pain assessment scales was used as applicable. The secondary outcomes were the dynamic composite pain score on gentle external and internal hip rotation which referred to rotating the hip gently until an obvious hip rotation was observed and usually involved 5 degrees of movement, and the tolerable hip flexion measured by a goniometer on post-intervention day 5 compared with those before neurolysis. The range of movement served as the functional outcome assessment. The percentage of patients who could achieve a satisfactory pain control, which is defined as a composite pain score 0–1 at hip flexion 80 degrees, was also evaluated. Interim outcomes on dynamic pain control were also evaluated in the outpatient clinic at post-intervention 4 to 6 months. Adverse events were defined as unfavourable and/or unintended findings (including abnormal laboratory results), or symptoms associated with the study procedure.

### 2.7. Data Analyses

The number of patients recruited in this retrospective study was reported. Wilcoxon signed rank test was used to compare the median dynamic composite pain scores with the baseline. T test was used to analyse the mean degree of tolerable hip flexion. SPSS version 26 (IBM Corp, Armonk, NY, USA) was used for analyses.

## 3. Results

A total of 74 patients were included in our study. Two patients were excluded because of incomplete data. The patient demographics are shown in Table 2 while the details of descriptive statistics are outlined in Table A1, Appendix A. Following anterior neurolysis, the dynamic pain at hip flexion 80° was significantly reduced from a median composite pain score of 3 at baseline to 1 on post-intervention day 5 (*p* < 0.001). The median composite pain score during gentle hip internal and external rotation reduced from 3 at baseline to 1 on post-intervention day 5 (*p* < 0.001) (Table A2, Appendix A). Functionally, the mean tolerable passive hip flexion degree significantly improved after hip neurolysis, from 39.7° at baseline to 74° on post-intervention day 5 (*p* < 0.001). The mean of differences between day 5 and baseline was 34.3° (95% CI, 28.8–39.8) (Table A3, Appendix A).

If we set our criterion of satisfactory pain control as a composite pain score ≤ 1 on hip flexion 80 degrees, 72% of the patients would meet this criterion. During the interim follow-up at 4 to 6 months after intervention, 47 patients attended and 45 of them reported satisfactory dynamic pain control (composite pain score ≤ 1) on hip flexion and gentle hip external and internal rotation. Among these 47 patients, 32 suffered from fracture neck of femur whilst 15 suffered from trochanteric fracture. The remaining patients either defaulted or passed away. The two patients who still suffered from significant hip pain were from the fracture neck of femur group.

We observed that around 50% of patients experienced a transient burning sensation which lasted for a few minutes after alcohol injection and then subsided spontaneously. However, we did not notice any recurrence of such burning sensations after the local anaesthetic effect wore off. There were seven patients (around 10%) who developed transient hypotension in the post-anaesthetic care unit (PACU) after the neurolytic procedure. All responded to fluid therapy and vasopressor treatments and could be discharged to the general ward afterwards. There was no report of lower limb sensory deficit as a result of femoral or obturator nerve block in all patients on post-intervention day 5. The overall 30-day mortality rate was 12.5%.

Regarding the IDEAL evaluation, this study was classified as stage 2a (development). Despite a retrospective nature of the study, it fulfilled the criteria of stage 2a (development) in terms of the number of patients according to a previous report implementing the IDEAL framework by Gerullis et al. [13]. It provided more data concerning the safety and efficacy of an established concept with the development of a modified approach.

## 4. Discussion

With the increasing elderly population globally, anterior hip neurolysis under ultrasound guidance has provided an important treatment option in the management of fragile hip fracture patients with severe comorbidities, and the results in our study were consistent with those in our first case series of anterior hip pericapsular neurolysis via the conventional approach [5]. Both the median composite dynamic pain score and the mean hip flexion range were markedly improved to a similar extent. In addition, over 70% of patients could enjoy satisfactory pain relief with movement, thus indicating that it is a reliable and effective technique in controlling the fracture pain in this challenging patient group. For the remaining 30% patients who responded suboptimally to anterior neurolysis, one of the postulations is the contribution and sensitization of the posterior hip capsule as supported by our recent case series on posterior hip pericapsular neurolysis [12]. Anatomically, this is explained by the presence of nociceptors in the superoposterior labrum, which is predominantly innervated by the nerve to the quadratus femoris with contributions from the superior gluteal and sciatic nerves [14,15,16]. The concept of silent nociceptor activation secondary to fracture hip can explain the augmented pain intensity over posterior hip capsule [17,18].

We arbitrarily selected 80 degrees as the cut-off for our hip flexion assessment because this degree of hip flexion would indicate the ability of sitting up comfortably. Pain control during hip rotation could reflect comfort in bed turning during nursing care. Since it usually takes several days for the full effects of alcohol neurolysis to be seen [19], post-intervention day 5 was chosen as the timing for primary and secondary outcome assessments. This would be an appropriate timing to indicate the long-term neurolysis outcome as well. As a number of patients would be discharged within a week provided that their pain control was satisfactory without active medical issue, it would be unethical and impractical to call them for follow-up assessments at a later time point. Regarding the finding of satisfactory pain control in the majority of patients (70%) on post-intervention day 5, we noticed that there were even more patients (45 out of 47 patients) who could achieve the same outcome 4 to 6 months after neurolysis. This might be explained by the subsidence of most painful pathological processes secondary to fracture healing by the time we followed up with them. All these results can further consolidate the benefits of anterior hip neurolysis in inoperable hip fracture regardless of trochanteric or neck fracture.

The two patients who persisted to have significant pain in long-term follow-up suffered from fracture neck of femur. It is well recognized that there was a 10–30% risk of non-union, subsequently avascular necrosis in femoral neck fracture despite surgery [20,21,22]. This can potentially attribute to the persistent pain in these two patients.

Motor assessment was an important area to illustrate in this motor-sparing technique. Nonetheless, it was challenging to objectively assess motor function in this group of patients since quite a few of them were cognitively impaired and there might have been significant pre-existing muscle wasting in some patients. Spontaneous lower limb movement might illustrate the integrity of motor function, but it was by all means non-specific to the femoral and obturator nerves. The disruption of lower limb mechanics as a result of fracture might also hinder lower limb movement. As such, we chose to use sensory testing by cold sensation as a surrogate to the integrity of the motor branch of the femoral and obturator nerves.

While the high FNAB branches consistently course over the periosteal surface of the pubis between the AIIS and IPE, the low FNAB branches are fewer in number and pierce the iliopsoas before reaching the iliofemoral ligament, and there is no relation to any sonographically identifiable landmark [23,24,25]. This explains why the low branches may be missed in a low volume injection between AIIS and IPE in the neurolysis setting, thus indicating the need for additional iliopsoas injection. One may argue if sole iliopsoas plane infiltration is sufficient to cover both high and low FNAB branches, as well as AON, and consequently, an additional PENG block may not be indicated [26]. Nonetheless, an obscured iliofemoral ligament secondary to the surrounding hematoma or disrupted femoral head may be occasionally seen. This may lead to significant difficulties in identifying and hydrodissecting the relevant structures. Conversely, a larger volume of injectate in the PENG plane, for instance 10 mL, may allow a spread to the iliopsoas plane to cover the low branch of FNAB. Yet, there is no guarantee, and any possible hematoma or bone spur around can also hinder such spread as well.

**Figure 8:**
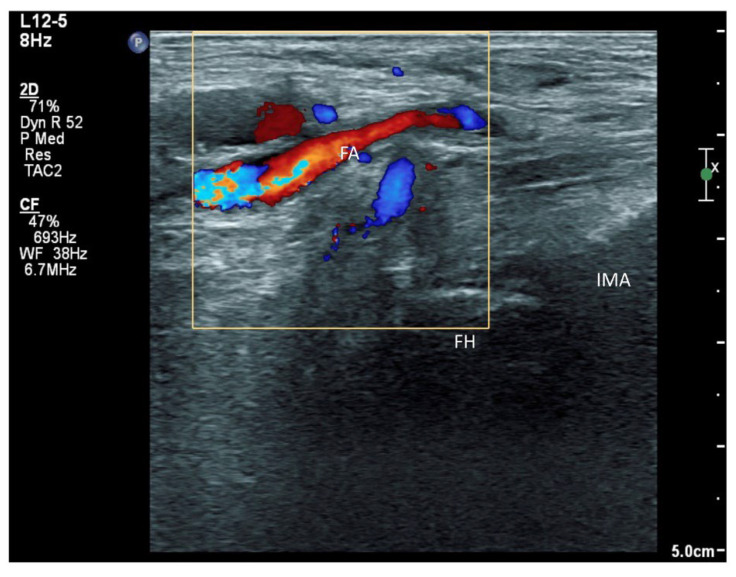
Doppler signal of the femoral artery and its branches when scanning the inferomedial acetabulum by the classical oblique approach. FH: femoral head, FA: femoral artery and branches, IMA: inferomedial acetabulum.

Our modified approach to the ONAB would have the following advantages over the ordinary oblique approach published in our first case series [5]. First, in patients with hip fracture, their fractured limb is commonly externally rotated. This would deviate the femoral vessels to overlay the needle trajectory to the ONAB at the inferomedial acetabulum (Figure 8). This sagittal approach can reliably avoid the femoral vessels in the trajectory. Second, the sagittal scan allows us to visualize the acetabular/posterior branch of the obturator vessels underneath the ONAB, and this can minimize the chance of inadvertent puncture of these small vessels. Third, the ONAB anatomically courses along the caudal aspect of IMA instead of the lateral aspect of IMA [24]. The modified approach can potentially target the ONAB more precisely. Lastly, the modified approach allows the ONAB to be visualized clearly in non-obese patients, whereas the ordinary approach only relies on a rough bony landmark of IMA. Although a large volume of subpectineal injection may lead to a spread to the motor branch of obturator nerve [27], we did not identify any case with neurological deficit along the obturator nerve distribution in our study.

Transient burning sensation immediately after alcohol injection is a potential pitfall of this treatment modality despite the prior local anaesthetic injection. According to our study, this side effect was transient and could be much minimized if we waited adequately after local anaesthetic injections. Prior explanation to patients could also facilitate tolerance to the transient discomfort. The absence of subsequent recurrence of burning sensation could be explained by the absence of skin innervation from the articular branches. More burning discomfort would result from absolute alcohol injection if a lower volume of local anaesthetics is injected beforehand. This, however, can yield a higher final alcohol concentration. It is commonly believed that a resultant concentration of at least 50% alcohol is required for a long-lasting neurolytic effect [28]. Regarding a possibility of variable alcohol concentrations owing to uneven mixing, the efficacy of this 1:1 ratio between local anaesthetic and absolute alcohol has been illustrated by this study and our first case series [5]. Alternatively, aqueous phenol has been shown to be effective in a tumour setting [29].

Since it was difficult to standardize perioperative analgesics in this group of patients and it was also common for them to have multiple analgesics for other pain conditions, perioperative analgesic consumption was not evaluated.

Methodology wise, we adopted a new reporting approach, the IDEAL framework that was proposed in 2009 by McCulloch et al. [30]. This descriptive framework provides clear stages of surgical innovation that allow every procedure or technique to be assigned to a particular level of development and evidence, based on factors such as the number of treated patients, the type of report, the study design and the aim of the report. Table 3 illustrates the five main stages of development according to the IDEAL criteria, with the respective specifications and requirements [13,30]. When summarizing all available publications concerning the technique of chemical denervation in inoperable hip fracture with the present study included [4,5,12], the number of inoperable fracture hip patients treated by this modality ranged from 1 to 74 patients, with a total of 165 patients. It is reasonable to comment that the technique is in the development stage (2a) according to this framework. The remaining IDEAL stages have not yet been met by the currently available evidence. However, it would be challenging to proceed to further stages. For instance, stage 3 (assessment) requires a randomized controlled trial with or without intervention. This could be logistically difficult for our patient subgroup, and it may also lead to ethical concerns. As suggested by Gerullis et al., if a surgical technique is reserved for a specific indication and patient subgroup, evidence of safety and efficacy, as required for stage 2a, may be accepted as sufficient and the highest quality level achievable [13]. Although a new treatment modality should desirably be classified in a prospective fashion according to IDEAL stage 2a, it is impossible to evaluate the status of all the surgical techniques currently reported in the literature in this way. Hence, retrospective analysis of the available data such as our study may be an appropriate and acceptable way to analyse the current IDEAL status of the current techniques regarding chemical hip neurolysis.

There are several limitations in this study. First, it was a retrospective case study, and this was subject to various biases. Second, the retrospective nature was not a desirable research format for the IDEAL classification stage 2a. Third, although the sagittal approach of targeting ONAB with a caudal-to-cephalad needle trajectory can avoid femoral vessels and ONAB can be better visualized, it is still unknown about the desired volume of absolute alcohol balancing the safety of obturator main trunk involvement and the maximum analgesic benefit. Other limitations included a lack of documentation of perioperative analgesic consumption, which might lead to biases in interpreting our composite pain score and a lack of formal testing of motor nerve integrity, such as a nerve conduction study that might be overlooked by simple clinical examination in cognitively impaired patients. Finally, it is also unknown about the desired injectate volumes for iliopsoas infiltration and PENG injection to faithfully cover all articular branches while minimizing the systemic side effects of absolute alcohol.

## 5. Conclusions

We conclude that anterior hip neurolysis by our novel modified approach (PENG approach, iliopsoas plane infiltration and sagittal approach to ONAB) provided satisfactory and consistent analgesic and functional benefits to the majority of patients with inoperable hip fractures. Compared with the classical approach (PENG approach and oblique approach to ONAB), this modified approach can offer a better safety profile theoretically. According to the IDEAL framework, despite some pitfalls, our study has shown the safety and stability of the modified anterior approach and can be classified as stage 2a. Complemented by our other retrospective case series on posterior hip pericapsular neurolysis [12], this chemical neurolysis technique for inoperable hip fracture would have been ready for the next stage of research in the IDEAL framework. This includes further evaluation focusing on defining the intervention, its indications, and the standards for acceptable quality of delivery via a collaborative prospective cohort study by multiple groups, including analysis of learning curves, followed by randomized controlled trials to evaluate the technique. Long-term data are also warranted to determine the long-term impact on mortality and morbidity compared with the conventional surgical treatment.

## Figures and Tables

**Table 1:** Composite pain scores to incorporate NRS, PAINAD and VDS.

Composite Pain Score	0 No Pain	1 Mild Pain	2 Moderate Pain	3 Severe Pain
NRS	0	1–3	4–6	7–10
PAINAD	0	1–3	4–6	7–10
VDS	No pain	Slight to mild pain	Moderate pain	Severe, very severe, and the most intense pain possible

**Table 2:** Patient demographics.

Characteristics	*n* (%)
Sex	
Female	46 (63.9)
Male	26 (36.1)
Mean age (years)	80.5
Type of femoral fracture	
Trochanter	24 (33.3)
Neck	48 (66.7)

**Table 3:** Stages of IDEAL classifications.

	Stage 1 Idea	Stage 2a Development	Stage 2b Exploration	Stage 3 Assessment	Stage 4 Long-Term Study
Purpose	Proof of concept	Development	Learning	Assessment	Surveillance
Patient number and types	Highly selected	Few; selected	Many; may expand to mixed; broadening indication	Many; expanded indications	All eligible
Surgeon number and types	Very few; innovators	Few; innovators and some early adopters	Many; innovators, early adopters	Many, early majority	All eligible
Output	Description	Description	Measurement; comparison	Comparison; complete information for non-RCT participants	Description; audit, regional variation; quality assurance; risk adjustment
Intervention	Evolving; procedure inception	Evolving; procedure development	Evolving; procedure refinement; community learning	Stable	Stable
Method	Case reports	Prospective development studies	Research database; explanatory or feasibility RCT; disease based	RCT with/without additions; alternative designs	Registry; routine data-base; rare-case reports
Outcomes	Proof of concept; technical achievement; disasters	Mainly safety; technical and procedural success	Safety; clinical outcomes; short-term outcomes; patient-centred and feasibility outcomes	Clinical outcomes; middle-term and long-term outcomes; patient-centred outcomes; cost effectiveness	Rare events; long-term outcomes; quality assurance
Ethics approval	Sometimes	Yes	Yes	Yes	No

## Data Availability

We provided the data in the Appendix A that we submit alongside the manuscript.

## Appendix A

**Table A1:** The descriptive statistics of resting and dynamic composite pain score (on hip flexion 80° and gentle hip rotation) and the maximally tolerable hip flexion degree before procedure and on post-procedural day 5.

	Preoperative Rest Pain	POD5 Rest Pain	Preoperative Dynamic Pain (Hip Flexion)	POD5 Dynamic Pain (Hip Flexion)	Preoperative Dynamic Pain (Hip Rotation)	POD5 Dynamic Pain (Hip Rotation)	Preoperative Hip Flexion ROM	POD5 Hip Flexion ROM
N Valid	72	72	72	72	72	72	72	72
Missing	0	0	0	0	0	0	0	0
Mean	0.67	0.17	2.65	1.00	2.82	1.53	39.65	73.96
Median	0.00	0.00	3.00	1.00	3.00	1.00	30.00	80.00
Minimum	0.00	0.00	0.00	0.00	1.00	0.00	0	20.00
Maximum	3.00	1.00	3.00	3.00	3.00	3.00	90	120.00

POD5: post-procedural day 5, ROM: range of movement, N: number of patients.

**Table A2:** The non-parametric test to analyse the median composite pain scores at rest/on hip flexion 80°/gentle hip rotation before procedure and on post-procedural day 5.

	Wilcoxon Signed Ranks Test		
	Rest Pain (Preoperative vs. POD5	Dynamic Pain (Hip Flexion) (Preoperative vs. POD5)	Dynamic Pain (Hip Rotation) (Preoperative vs. POD5)
Z	−4.54 ^a^	−6.976 ^a^	−6.280 ^a^
*p*-value	<0.001	<0.001	<0.001

^a^ Based on negative ranks; Z: Z score; POD5: post-procedural day 5.

**Table A3:** The parametric test to analyse the mean maximally tolerable hip flexion before procedure and on post-procedural Day 5.

Paired Samples Test									
		Paired Differences			95% Confidence Interval of the Difference				
		Mean	Standard Deviation	Standard Error of Mean	Lower	Upper	*t*	df	*p*-Value
Pair 1	Hip flexion ROM (POD5 vs. preoperative)	34.30556	23.57645	2.77851	28.76536	39.84575	12.347	7112.347	<0.001

POD5: post-procedural day 5; ROM: range of movement; df: degrees of freedom, *t*: the test statistic for the paired *t*-test.
